# Skin immunization for effective treatment of multifocal melanoma refractory to PD1 blockade and Braf inhibitors

**DOI:** 10.1136/jitc-2020-001179

**Published:** 2021-01-06

**Authors:** Xingxing Hao, Louis D Falo III, Guo Chen, Jiying Zhang, Cara D Carey, Walter J Storkus, Louis D Falo Jr, Zhaoyang You

**Affiliations:** 1 Department of Dermatology, University of Pittsburgh School of Medicine, Pittsburgh, Pennsylvania, USA; 2 Dietrich School of Arts & Sciences, University of Pittsburgh, Pittsburgh, Pennsylvania, USA; 3 Department of Immunology, University of Pittsburgh School of Medicine, Pittsburgh, Pennsylvania, USA; 4 Department of Pathology, University of Pittsburgh School of Medicine, Pittsburgh, Pennsylvania, USA; 5 The University of Pittsburgh Medical Center Hillman Cancer Center, Pittsburgh, Pennsylvania, USA; 6 Department of Bioengineering, University of Pittsburgh Swanson School of Engineering, Pittsburgh, Pennsylvania, USA; 7 The University of Pittsburgh Clinical and Translational Science Institute, Pittsburgh, Pennsylvania, USA; 8 The University of Pittsburgh McGowan Institute for Regenerative Medicine, Pittsburgh, Pennsylvania, USA

**Keywords:** vaccination, immunotherapy, melanoma

## Abstract

**Background:**

Despite the remarkable benefits associated with the interventional treatment of melanomas (and other solid cancers) with immune checkpoint and Braf inhibitors (Brafi), most patients ultimately progress on therapy. The presence of multifocal/disseminated disease in patients increases their mortality risk. Hence, the development of novel strategies to effectively treat patients with melanomas that are resistant to anti-PD1 mAb (αPD1) and/or Brafi, particularly those with multifocal/disseminated disease remains a major unmet clinical need.

**Methods:**

Mice developing induced/spontaneous Braf^V600E^/Pten^−/−^ melanomas were treated by cutaneous immunization with a DNA vaccine encoding the melanoma-associated antigen TRP2, with Brafi or αPD1 alone, or with a combination of these treatments. Tumor progression, tumor-infiltration by CD4^+^ and CD8^+^ T cells, and the development of TRP2-specific CD8^+^ T cells were then monitored over time.

**Results:**

Vaccination led to durable antitumor immunity against PD1/Brafi-resistant melanomas in both single lesion and multifocal disease models, and it sensitized PD1-resistant melanomas to salvage therapy with αPD1. The therapeutic efficacy of the vaccine was associated with host skin-resident cells, the induction of a systemic, broadly reactive IFNγ^+^CD8^+^ T cell repertoire, increased frequencies of CD8^+^ TIL and reduced levels of PD1^hi/int^CD8^+^ T cells. Extended survival was associated with improved TIL functionality, exemplified by the presence of enhanced levels of IFNγ^+^CD8^+^ TIL and IL2^+^CD4^+^ TIL.

**Conclusions:**

These data support the use of a novel genetic vaccine for the effective treatment of localized or multifocal melanoma refractory to conventional αPD1-based and/or Brafi-based (immune)therapy.

## Introduction

Immune checkpoint PD1 mAb (anti-PD1 mAb (αPD1)) and oncogene Braf inhibitors (Brafi) represent effective treatment options for patients with melanoma or other solid cancers,^1 2^ but most treated patients ultimately exhibit disease progression. Intrinsic or acquired tumor resistance to these modalities is commonly observed in the clinic, limiting their overall utility.^3 4^

Brafi alter the tumor microenvironment (TME) by enhancing tumor antigen (Ag) expression, T cell recruitment and local production of Interferon gamma (IFNγ), while coordinately reducing immune suppression.^5–8^ Brafi-resistant tumors typically exhibit defects in tumor Ag presentation and CD8^+^ tumor-infiltrating lymphocyte (TIL) numbers/function.^9–11^ Administration of αPD1 can reinvigorate ‘exhausted’ CD8^+^ T cells leading to improved antitumor activity and therapeutic benefits.^12–14^ The importance of CD4^+^ T cells in this revolutionary treatment paradigm is also emerging.^15–22^ PD1 refractory disease is generally associated with a TME characterized by: (1) increased prevalence of immune suppressor cells; (2) defects in Ag cross-presenting CD103^+^/XCR1^+^ dendritic cells (DCs); (3) limited stromal expression of CXCR3 ligand chemokines; (4) lack of IFNγ signaling; (5) reduced PDL1 and MHC I/II expression; and (6) sparse CD8^+^ TIL content, among others.^1 3 12–14 23^

A range of interventional strategies (eg, vaccines, costimulatory agonists, intratumoral-delivered oncolytic herpes virus, adoptive cell therapies, radiation/chemotherapy, targeted regulatory antagonists, modulators of metabolism or gut microbiome, among others) have been explored to render cancers more responsive to αPD1 or Brafi treatment.^9 11 23–36^ Theoretically, specific immunization is an ideal salvage approach for overcoming αPD1/Brafi-resistance due to its potential to generate broadly reactive tumor-specific CD8^+^ T cells that are capable of attacking disseminated disease directly while coordinately reconditioning the immunologically ‘cold’ and/or immunosuppressive TME for improved immune responsiveness and receptivity to intervention immunotherapies, including αPD1 or Brafi.^36–43^

Spliced x-box binding protein 1 (xbp1)—an endoplasmic reticulum stress-associated factor—functions as a ‘master’ transcription factor in regulating expression of a broad spectrum of genes in a cell context-specific manner.^44 45^ For instance, in an ovarian cancer model, xbp1 expression is associated with the activation of immunosuppressive pathways,^46 47^ while its activity in immune cells is linked to protective responses against viral infection, potentiation of cutaneous autoimmune disease, activation of NK cells and macrophages, and the differentiation, survival and function of DC subsets, including plasmacytoid DC (pDC) and CD103^+^ DC.^48–56^ We and others have reported the importance of intrinsic xbp1 within DC for the promotion of robust antitumor immune responses.^57–59^ Since heat shock protein 70 (hsp70) can be used as a carrier for targeted Ag delivery and as an ‘adjuvant’ for DC maturation to improve immune responses,^60^ ectopic gene approaches coordinately driving xbp1 and hsp70 expression (via gene therapy) have potential to further enhance the therapeutic efficacy of cancer vaccines. Indeed, we previously determined that skin immunization with cDNA encoding xbp1 and hsp70 fused to a tumor-associated antigen is therapeutic against established tumors in a CD103^+^DC-dependent, pDC-dependent and CD8^+^ T cell-dependent manner.^57 58^

In the current report, we demonstrate that this genetic vaccine approach serves as an effective salvage therapy for multifocal Braf^V600E^Pten^−/−^ melanomas exhibiting resistance to PD1 blockade and Brafi by promoting durable antitumor immunity and resensitizing these melanomas to treatment with αPD1. The therapeutic efficacy of this interventional approach was dependent on skin-resident cells and associated with the induction of broadly reactive IFNγ^+^CD8^+^ T cell responses in the periphery and the improved fitness/function of CD4^+^ and CD8^+^ TIL.

## Results

### Skin immunization but not Brafi effectively treats tamoxifen-induced Braf^V600E^Pten^−/−^ melanomas that are intrinsically resistant to anti-PD1 checkpoint blockade

**Figure 1:**
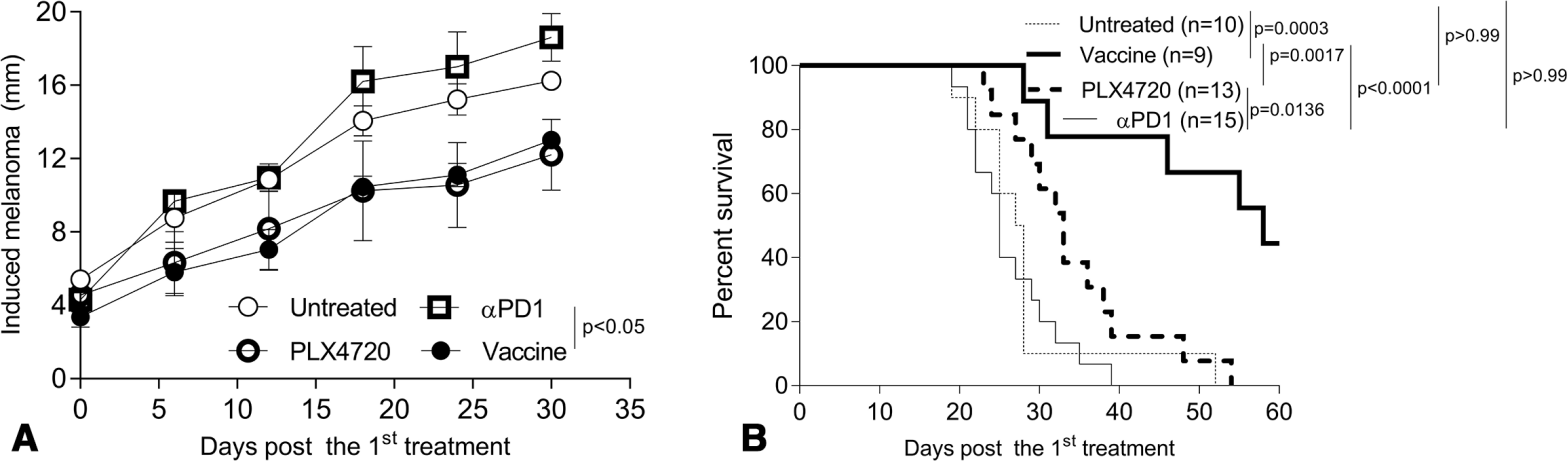
Skin immunization but not Brafi treatment elicits durable antitumor immunity against PD1-resistant, induced melanomas. Induced melanomas were developed by topical treatment of skin with tamoxifen in B6-Tyr-Cre^ERT2^Braf^CA^Pten^lox/lox^ mice. Melanoma-bearing mice were randomized into cohorts of three to five mice exhibiting comparable mean aggregate tumor size. Mice were then left untreated or they were treated (the day of first treatment was defined as day 0) by skin immunization using a GG on days 0, 7 and 14, oral administration of Brafi PLX4720 daily for 10 days or intraperitoneal injection with αPD1 mAb every other day × 4. Tumor growth and animal survival were monitored every 3 days. Data from three experiments are depicted and were statistically analyzed using a Student’s t-test (tumor size) or a log-rank test (animal survival). Mice developing spontaneous melanomas were excluded from these analyzes. αPD1, anti-PD1; Brafi, Braf inhibitors; GG, gene gun; n, numbers of mice used in experiments.

Gene gun (GG)-mediated cutaneous immunization with cDNA encoding xbp1 and hsp70 fused to the melanoma-associated antigen TRP2 elicits durable antitumor immunity against Braf^V600E^/Pten^−/−^ melanomas (figure 1).^58^ Consistent with findings from others,^61^ we observed that the conditionally induced melanomas were intrinsically resistant to PD1 blockade monotherapy (figure 1). In this model, we and others noted that melanomas also exhibited either intrinsic-resistance or acquired-resistance to Brafi (ie, PLX4032 and PLX4720) treatment (data not shown).^62^ Although Brafi-based therapy delayed the growth of Brafi-responsive melanomas initially (figure 1A), these effects dissipated on treatment discontinuation (figure 1B), suggesting the transience of the protective antitumor response promoted by Brafi treatment. Remarkably, skin immunization was effective in controlling tumor growth regardless of intrinsic/acquired Brafi-resistance status, resulting in durable antitumor immunity (figure 1A, B).

### Skin immunization renders PD1 checkpoint refractory melanoma responsive to αPD1-based therapy

**Figure 2:**
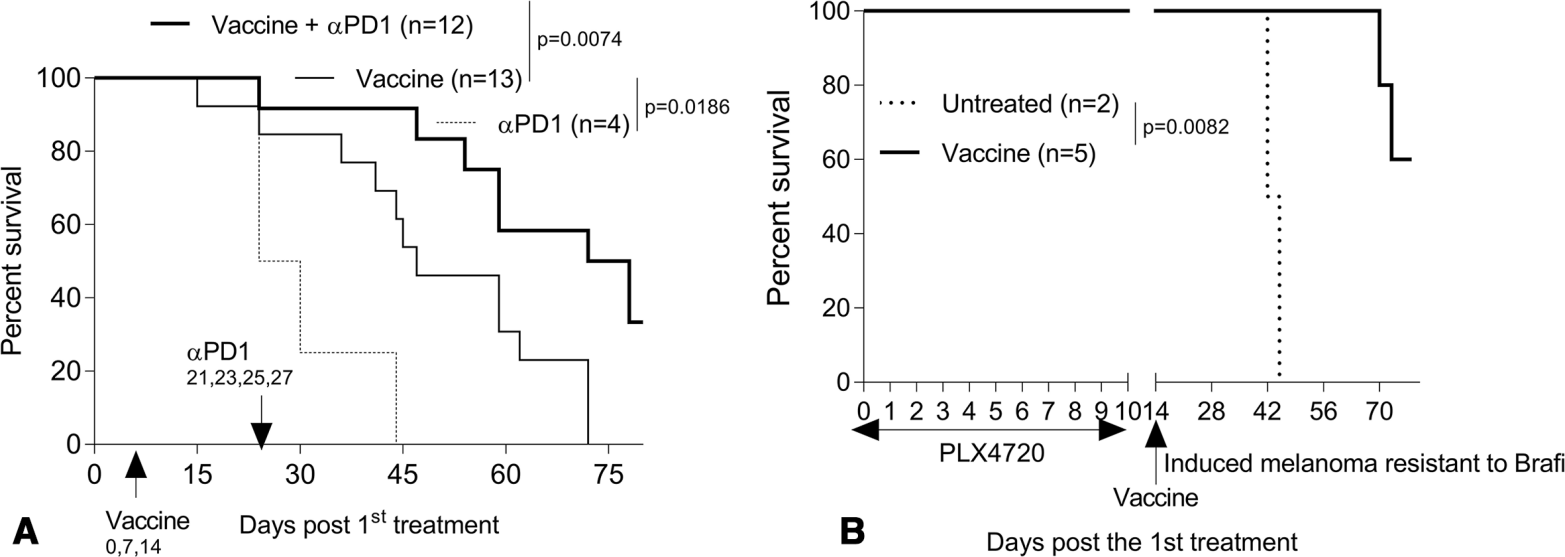
Skin immunization renders PD1-resistant, tamoxifen-induced melanoma responsive to αPD1 mAb therapy (A). Induced melanoma-bearing mice were left untreated or they vaccinated weekly three times as described in figure 1, with a portion of vaccinated mice then administered αPD1 mAb every other day × 4, as indicated. Brafi-resistant, tamoxifen-induced melanomas respond to skin immunization (B). Mice bearing tamoxifen-induced melanomas were treated daily with Brafi PLX4720 on days 0–9. On day 14, mice bearing PLX4720-resistant melanoma were then left untreated or they received a single skin immunization. (A and B) Animal survival was monitored every 3 days. Data from three (A) to two (B) independent experiments are shown and statistically analyzed. Mice bearing spontaneous melanomas were excluded from the analyzes. αPD1, anti-PD1; Brafi, Braf inhibitors; n, numbers of mice used in experiments.

Since the induced Braf^V600E^/Pten^−/−^ melanomas were intrinsically resistant to PD1 checkpoint blockade^61^ (figure 1), we next investigated whether skin immunization renders these melanomas responsive to treatment with αPD1 mAb. Mice bearing induced melanomas were vaccinated on a weekly basis for three consecutive weeks. One week after the last immunization, mice were randomized and left untreated, or they were administered αPD1 mAb (intraperitoneal) every other day × 4. As shown in figure 2A, treatment with the vaccine + αPD1 mAb significantly improved the overall survival of mice bearing intrinsically PD1-resistant melanomas.

### Brafi-resistant, tamoxifen-induced melanomas respond to skin immunization

Despite the intrinsic resistance of tamoxifen-induced melanomas to Brafi, Brafi promotes a proinflammatory TME, as evidenced by increased tumor Ag expression, TIL recruitment, local production of IFNγ and decreasing immune suppression.^5–8^ We therefore examined whether Brafi-resistant tumors would respond to skin immunization. Mice bearing tamoxifen-induced melanomas were treated daily with Brafi PLX4720 on days 0–9. On day 14, mice bearing PLX4720-resistant melanoma were left untreated or they received a single skin immunization. As shown in figure 2B, Brafi treatment-resistant melanomas were responsive to treatment using skin immunization.

### Skin immunization but not Brafi monotherapy effectively treats multifocal disease in the Braf^V600E^/Pten^−/−^ melanoma model

Multifocal/disseminated melanomas have a poor clinical prognosis; hence, there is great need to develop effective treatment strategies for this patient population. Approximately half of all Braf^V600E^/Pten^−/−^ mice spontaneously develop melanomas even in the absence of tamoxifen induction (data not shown).^63^ Hence, after tamoxifen induction, two cohorts of melanoma-bearing mice develop: (1) an ‘induced’ group exhibiting tumors only at the site of tamoxifen treatment and (2) a ‘multifocal’ disease group exhibiting tumors at the site of tamoxifen treatment and additional tissue sites involving development of ‘spontaneous’ tumors (online supplemental figure 1).

10.1136/jitc-2020-001179.supp1 Supplementary data

**Figure 3:**
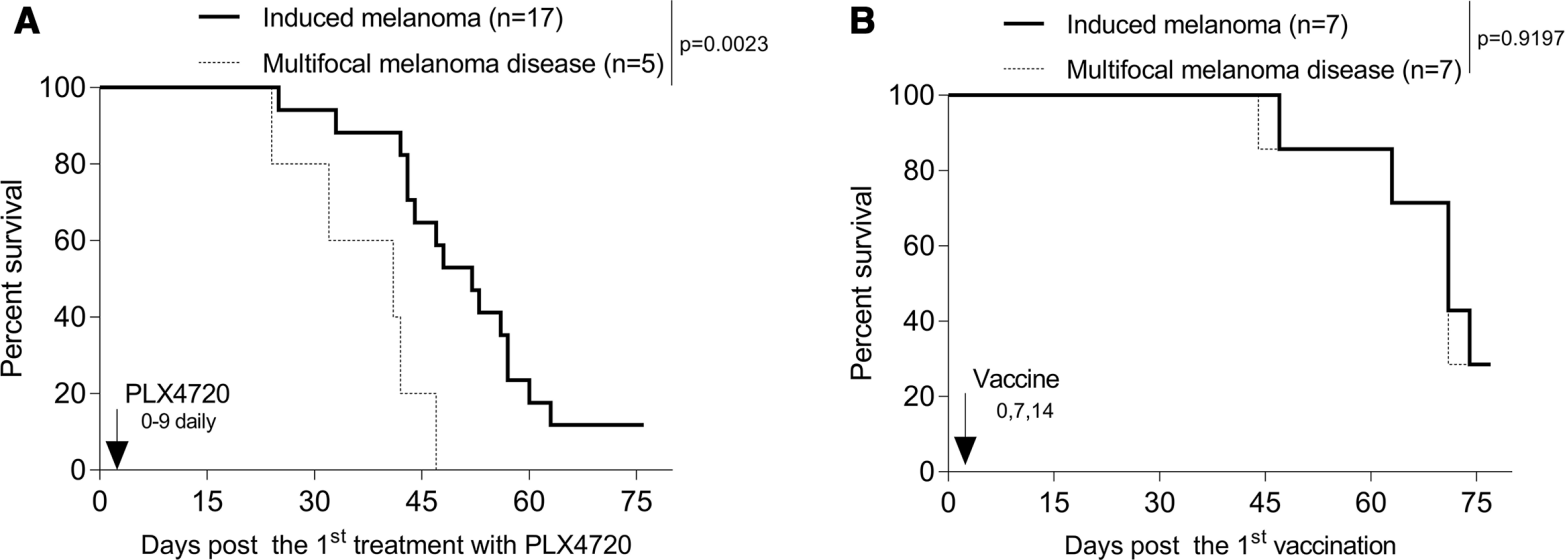
Skin immunization but not Brafi therapy effectively treats mice with multifocal melanoma disease. Mice bearing tamoxifen-induced melanomas were left untreated or they were treated with Brafi (A) or skin immunization (B) as described in figure 1. Animal survival were monitored every 3 days. Data from two independent experiments are shown and statistically analyzed. Multifocal melanoma disease was determined as mice bearing melanomas localized to sites of tamoxifen application+melanomas at distal non-induction sites (A and B). Mice bearing induced melanoma resistant to Brafi therapy were excluded from the analyzes (A). Brafi, Braf inhibitors; n, numbers of mice used in experiments.

To determine the differential impact of skin immunization Braf^V600E^/Pten^−/−^ mice bearing induced versus multifocal disease, these cohorts were treated with the vaccine or Brafi. As shown in figure 3A, Brafi treatment of the induced cohort of mice, but not multifocal disease cohort, resulted in a slight extension of overall animal survival. In contrast, skin immunization equitably extended overall survival in both cohorts of animals (figure 3B).

### Prophylactic skin immunization controls tamoxifen-induced melanoma development in Braf^V600E^/Pten^−/−^ mice and sensitizes mice with multifocal disease to αPD1-based immunotherapy

**Figure 4:**
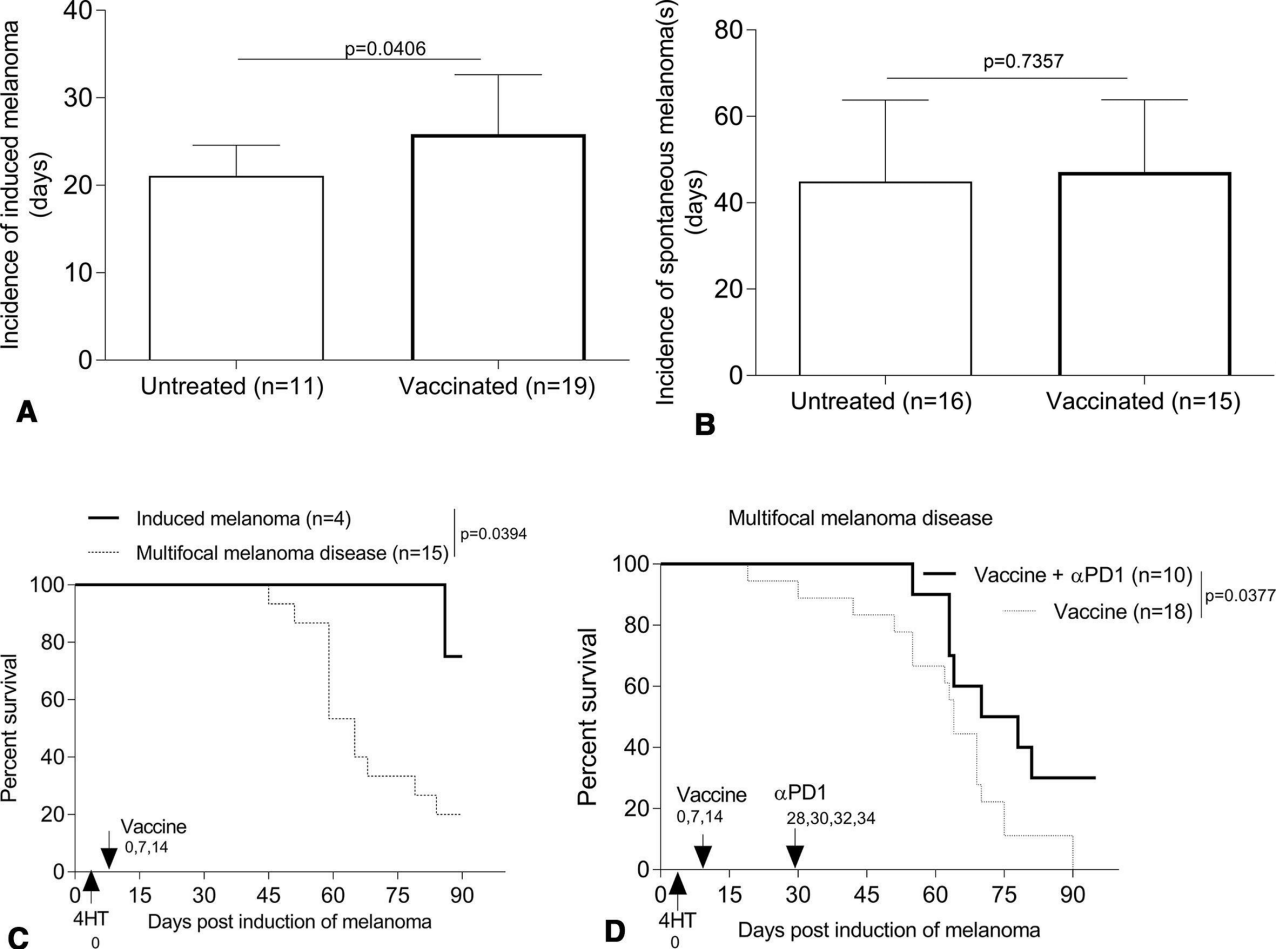
Prophylactic skin immunization controls development of tamoxifen-induced melanoma and renders intrinsically αPD1-resistant multifocal melanoma disease responsive to PD1 blockade. B6-Tyr-Cre^ERT2^Braf^CA^Pten^lox/lox^ mice (male/female, 3–4 weeks) were skin vaccinated three times on a weekly basis (days 0, 7 and 14, with the first day of treatment was defined as day 0). On day 0, mice received topical tamoxifen for induction of melanoma. Some of vaccinated-mice were administered αPD1 every other day x 4, as indicated in figure (D). Induced/spontaneous melanoma incidence and growth and overall survival of animals were monitored every 3 days. Data from two to four experiments are depicted and statistically analyzed using Student’s t-test (A and B) or Log-rank test (C and D). Mice lacking spontaneous melanoma were excluded from the analyzes (D). αPD1, anti-PD1; n, numbers of mice used in experiments.

To examine the ability of skin immunization to prevent the development of tamoxifen-induced melanomas or multifocal disease in Braf^V600E^/Pten^−/−^ mice, animals were first vaccinated, then left untreated or treated topically with tamoxifen to induce local tumor formation. Induced/spontaneous melanoma incidence and growth and the overall survival of animals were then monitored over time. Prophylactic vaccination significantly but only slightly reduced the incidence of tamoxifen-induced melanoma development (figure 4A) but had minimal impact on the spontaneous melanomas developing distal to sites of tamoxifen application (figure 4B). Skin immunization also appeared to preferentially control the growth of the induced but not multifocal melanomas and only extended the survival of mice bearing melanomas restricted to sites of tamoxifen induction (figure 4C).

To determine whether prophylactic skin immunization renders (intrinsically αPD1 resistant) multifocal melanoma disease responsive to subsequent αPD1 treatment, mice were cutaneously vaccinated, with tamoxifen then applied topically to induce melanomas on day 0. Skin immunization was repeated twice on days 7 and 14. On day 28 when conditionally induced melanomas were detected in the skin, αPD1 was administered every other day × 4. In the setting of multifocal melanoma disease, mice receiving both skin immunization + αPD1 treatment survived longer than mice receiving only skin immunization (figure 4D).

### Skin immunization improves therapeutic efficacy over DC cell-based therapies

We previously reported that therapeutic efficacy of skin immunization depends on: (1) skin migratory CD103^+^ DC in transplantable mouse models of melanoma, breast carcinoma and glioma and (2) systemic CD8^+^ T cell responses.^58^ We also demonstrated that ex vivo-generated bone marrow (BM)-derived DC genetically engineered with our cDNA vaccine construct serve as an effective therapeutic agent after intraperitoneal delivery against transplanted subcutaneous 4T1.2-Neu breast carcinomas and GL26 gliomas,^58^ a finding that we have also recently corroborated in subcutaneous GL26 glioma models (online supplemental figure 2).

**Figure 5:**
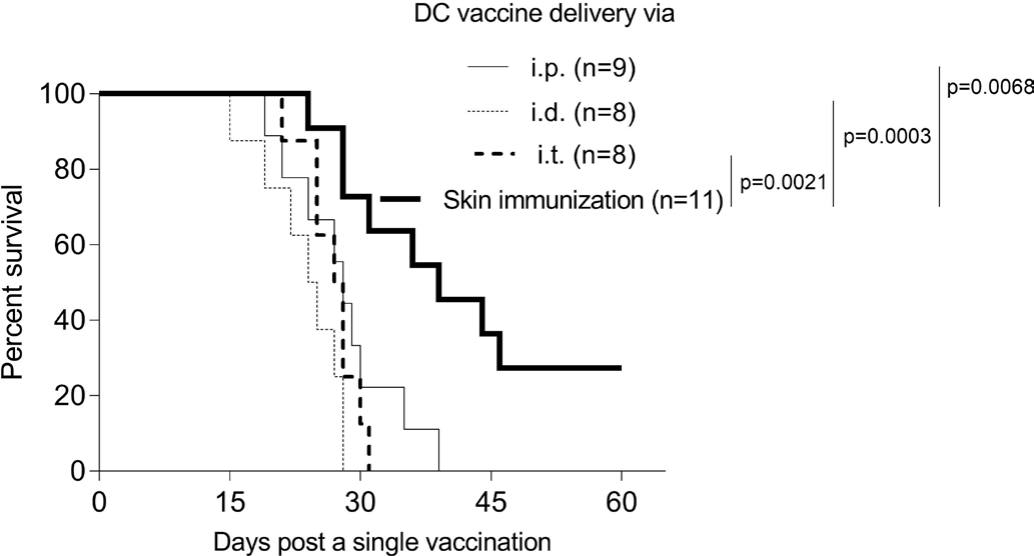
Skin immunization mediates superior antitumor efficacy versus DC-based genetic immunization. BM-derived DC engineered with vaccine cDNA served as the DC-based vaccine. Mice solely bearing tamoxifen-induced melanomas received a single skin immunation (figure 1) or a single DC-based vaccine via intraperitoneal, i.d. or intratumoral delivery. Animal survival was monitored every 3 days. Data from two independent experiments are depicted and statistically analyzed. BM, bone marrow; DC, dendritic cells; n, numbers of mice used in experiments.

To compare the therapeutic potential of skin-targeted versus adoptively transferred DC genetic vaccines in our Braf^V600E^/Pten^−/−^ melanoma models, mice bearing tamoxifen-induced melanomas were treated a single time with skin immunization or the DC/genetic vaccine delivered intraperitoneally, intradermal (i.d.) or intratumorally. Remarkably, the sustained therapeutic benefits observed in mice receiving the skin immunization could not be replicated using any of the DC-based vaccine approaches (figure 5), suggesting the superior (bio)efficacy of vaccine-accessed skin resident cells including dermal migratory CD103^+^ DC^58^ in the observed therapeutic benefits associated with skin immunization.

### The therapeutic efficacy of skin immunization relies on broadly reactive CD8^+^ T cells and their extended survival/persistence in the TME

**Figure 6:**
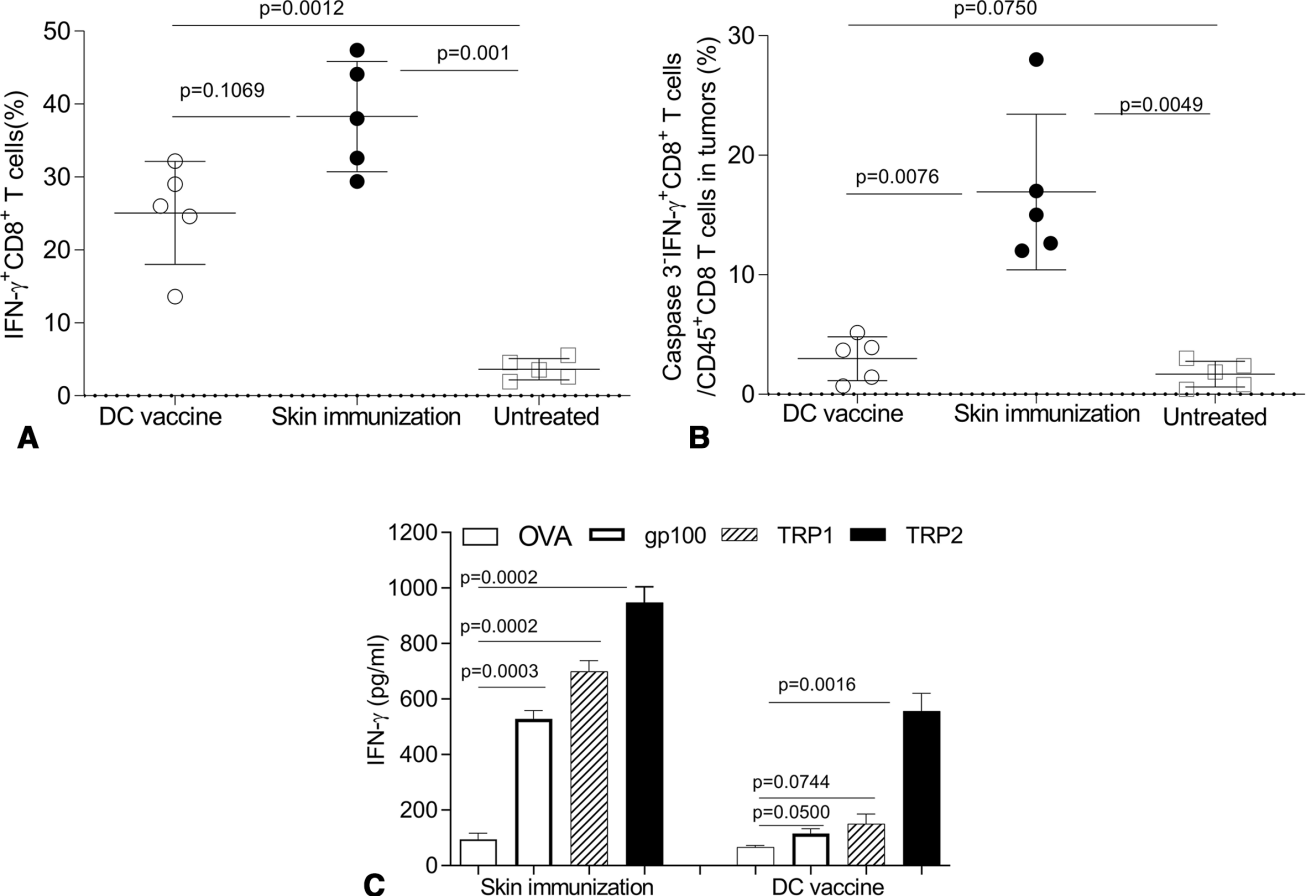
The therapeutic efficacy of skin immunization is associated with a broadly reactive CD8^+^ T cell response and enhanced CD8^+^ T cell fitness in the TME. Mice bearing induced melanomas were left untreated or they were treated once by the skin immunization or the DC/genetic immunization (as in figure 5). Twenty-four days later, mice were intravenously injected with brefeldin A, and after 4 hours, single cells of skin tdLN (A) were stained with anti-CD8 on the cell surface and stained intracellularly for IFNγ. Single cells from tumor enzymatic digests (B) were stained with Fixable Viability Dye (for excluding dead cells), anti-CD45, anti-CD8, anti-IFNγ and anti-Caspases 3 and analyzed by flow cytometry. IFNγ^+^CD8^+^ T cells in total CD8^+^ T cells of tdLN and intratumoral Caspases3^−^IFNγ^+^CD8^+^ T cells among live CD45^+^CD8^+^ gated cells from five mice from independent experiments are reported and statistically analyzed. In some experiments, splenocytes recovered from mice not receiving preparative injection of BFA were restimulated with peptides: mouse TRP2_180–188_, gp100_25-33_, TRP1_222-229_ or OVA_257-264_ for 3 days (C). The concentration of IFNγ in the culture supernatants was then measured by ELISA. Data from three independent experiments are depicted and statistically analyzed. DC, dendritic cells; tdLN, tumor-draining lymph node.

To better understand mechanism(s) underlying treatment benefits associated with skin immunization, we monitored IFNγ^+^CD8^+^ T cell frequencies in the skin tumor-draining lymph nodes (tdLN) of mice receiving skin versus DC-based genetic immunization. Although the DC-based vaccine failed to protect mice (figure 5), substantial numbers of IFNγ^+^CD8^+^ T cells were identified in tdLN receiving either immunization regimen (with no significant difference between groups but a trend towards superiority of skin immunization; figure 6A).

These data suggested that the magnitude of the vaccine-induced IFNγ^+^CD8^+^ T cell response was not a discriminator for differential vaccine antitumor efficacy. Indeed, recent reports suggest that susceptibility to apoptosis among CD8^+^ TIL restricts their therapeutic effectiveness.^64 65^ To examine the differential fitness of CD8^+^ TIL in mice treated by the skin versus DC-based genetic immunization, apoptotic Caspase 3 negative (Caspase 3^−^) IFNγ^+^CD8^+^ TIL were monitored post-treatment by flow cytometry. While a comparable increase of CD8^+^ T cells was observed in mice treated with either vaccine (data not shown), skin immunization preferentially resulted in accumulation of ‘healthier’ Caspase 3^−^IFNγ^+^CD8^+^ TIL versus DC/genetic immunization (figure 6B).

Others have questioned the ability of single Ag-based vaccines to elicit a broadly reactive CD8^+^ T cell repertoire capable of mediating therapy benefits against antigenically heterogeneous populations of tumor clones in the setting of visceral disease.^41 43^ To determine whether skin immunization leads to Ag spreading in the antimelanoma CD8^+^ T cell repertoire, single-cell suspensions of spleens were isolated from treated mice and then restimulated in vitro with MHC class I-presented peptide epitopes derived from vaccine Ag TRP2 or non-vaccine (but melanoma-associated) Ags gp100 and TRP1. An H-2K^b^-presented peptide epitope derived from ovalbumin (OVA) was used as an irrelevant specificity control. As expected, both immunization protocols resulted in the expansion of TRP2-specific CD8^+^ T cells. However, only CD8^+^ T cells from mice treated by skin immunization recognized the non-vaccine melanoma Ags gp100 and TRP1 (figure 6C).

When taken together, these data suggest the improved operational fitness of TRP2-specific TIL after skin immunization leads to corollary rounds of T cell cross-priming in tdLN, culminating in Ag-spreading in the therapeutic CD8^+^ T cell repertoire association with superior treatment outcome.

### Therapeutic efficacy of skin immunization is associated with superior CD4^+^ and CD8^+^ T cell infiltration in the TME

**Figure 7:**
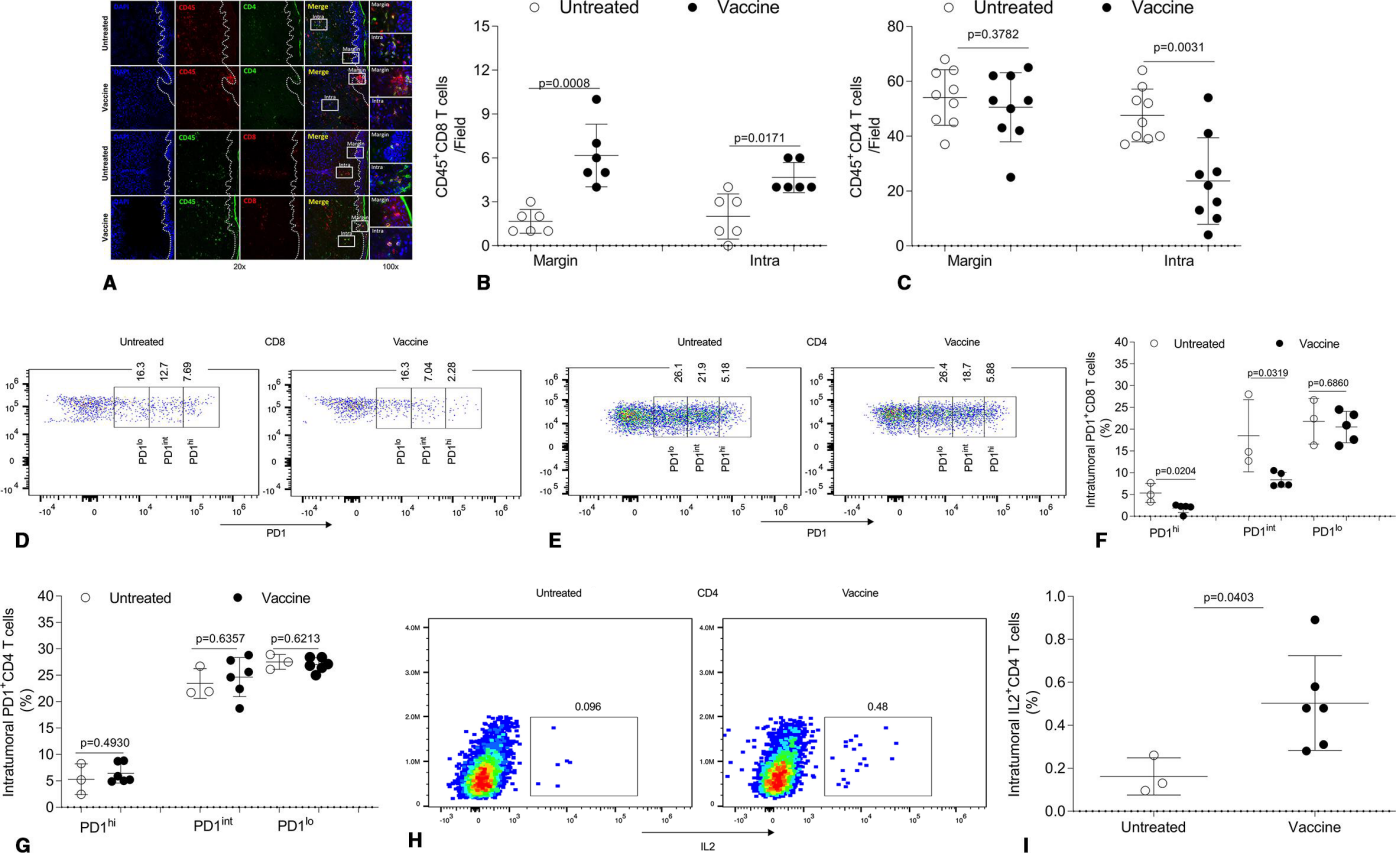
Therapeutic efficacy of skin immunization is associated with a favorable tumor infiltration by CD4^+^ and CD8^+^ TIL with improved fitness/functionality. Mice bearing induced melanoma were left untreated or they were treated by skin immunization (as described in figure 1). Two weeks after the last immunization, a portion of the tumor was frozen and analyzed by IF. One representative of three independent experiments using three (untreated) to six (treated) mice yielding similar results depicts CD45^+^CD4^+^ and CD45^+^CD8^+^ T cells at the margin and in the tumor core (A). Numbers of CD45^+^CD4^+^ and CD45^+^CD8^+^ T cells at the margin and intratumor per field from three independent experiments in (A) are quantified and statistically analyzed (B and C). At the time of harvest, tumor tissues were also digested and resultant single cells cultured in the presence of brefeldin A. After 4 hours, the cells were stained with anti-CD45, anti-CD8, anti-CD4 and anti-PD1 (surface) and anti-IL2 (intracellular) and subsequently analyzed by flow cytometry. Dead cells were stained with Fixable Viability Dye eFluor780. One representative result from three independent experiments performed using three to six mice depicts PD1^+^CD8^+^ TIL (D), PD1^+^CD4^+^ TIL (E), and IL2^+^CD4^+^ TIL (H). Frequencies of cells expressing PD1 high (PD1^hi^), intermediate (PD1^int^) or low (PD1^lo^) among CD4^+^ or CD8^+^ T cells in the live CD45 gate (FVDeFluor780^−^CD45^hi^) (F and G) and of IL2^+^CD4^+^ TIL in the live CD45 gate (FVDeFluor780^−^CD45^hi^) (I) are shown and statistically analyzed. IF, immunofluorescence; TIL, tumor-infiltrating lymphocyte.

To further understand mechanism(s) for the superior bioefficacy of skin immunization, we focused on treatment-evoked changes in tumor-infiltrating CD8^+^ T cells and CD4^+^ T cells, which have recently attracted attention in the context of effective αPD1-based immunotherapy.^15–22^ Skin immunization increased tumor-infiltration by CD8^+^ T cells in both the tumor margin and core (figure 7A, B), consistent with observed IFNγ^+^CD8^+^ TIL persistence (figure 6C). Skin immunization also resulted in fewer CD8^+^ TIL cells expressing a high PD1 (PD1^hi^) phenotype (figure 7D, F), which has been linked to poor prognosis in patients with cancer.^66–68^ Although skin immunization did not result in a change in numbers of CD4^+^ T cells at the tumor margin, it resulted in significantly decreased numbers of CD4^+^ TIL in the tumor core (figure 7A, C). Unlike their CD8^+^ counterparts, these latter CD4^+^ TIL failed to exhibit a reduction in PD1 expression (figure 7E, G). In our melanoma model, large numbers of intratumoral CD4^+^ T cells are Foxp3^+^CD4^+^ T cells (Treg),^62^ which exhibit enhanced suppressive activity.^69^ Thus, the reduced levels of CD4^+^ TIL in the tumor core of animals receiving skin vaccination could reflect fewer Treg and a less immunosuppressive TME. Furthermore, accumulated evidence suggests that IL2 signaling in the TME favors responsiveness to PD1 blockade.^70–73^ As shown in figure 7H, I, skin immunization resulted in the recruitment of increased levels of IL2^+^CD4^+^ TIL in the tumor core, which may at least partially explain the ability of skin immunization to convert PD1-resistant tumors into αPD1-responsive tumors (figures 2A and 4D).

## Discussion

Melanoma resistance to current frontline treatment regimens, including Brafi and checkpoint inhibitors, remains an impediment to achieving durable objective clinical responses in most patients. Consensus views suggest that such treatment resistance may be overcome via conditioning regimens/vaccines that promote the induction and maintenance of tumor-specific CD8^+^ T cell responses in vivo, particularly those within the TME (ie, CD8^+^ TIL).

Although skin is considered a highly immunogenic organ and a seemingly ideal anatomical target for immunization,^74 75^ most cancer vaccines introduced into the dermis have yielded minimal clinical benefit. We have recently developed a skin genetic vaccine platform enabling local expression of xbp1/hsp70 and tumor Ag cDNA, which effectively controls tumor growth in a skin CD103^+^ DC-dependent, pDC-dependent and CD8^+^ T cell-dependent manner in diverse mouse tumor models.^57 58^ In the current report, we provide novel findings supporting the ability of this vaccine strategy to overcome tumor resistance to Brafi and/or PD1 checkpoint blockade in multifocal disease models of melanoma reflective of patients with multiple primary disease sites/disseminated disease.

In the tamoxifen-induction model, developing melanomas are poorly infiltrated by CD8^+^ TIL (61, figures 6B and 7A, B), which may explain their observed intrinsic resistance to treatment with αPD1 (figure 1), known to require a proinflammatory TME at baseline.^61^ Remarkably, we observed that mice bearing αPD1-resistant melanomas when treated with skin immunization became responsive to subsequent αPD1 therapy (figure 2A). We believe that this may be due to the ability of skin immunization to facilitate tumor-infiltration and the enhanced fitness of responder CD8^+^ TIL, resulting in their extended survival and antitumor functionality within the TME (figures 6B and 7A, B, D, F). Since frequencies of IFNγ^+^CD8^+^ T cells in tdLN isolated from mice receiving skin versus DC-based genetic immunization were comparable (figure 6A), the inferior therapeutic benefit associated with the latter approach likely reflects the inability of treatment-induced T cells to mediate sustained effector function within the TME.

Recent data support intratumoral T cell apoptosis as a mechanism restricting the antitumor immune protection of the host.^64 65^ Indeed, we noted a superior fitness phenotype for IFNγ^+^CD8^+^ TIL (Caspase 3^−^ IFNγ^+^CD8^+^ T cells) in mice treated by skin versus DC-based genetic immunization (figure 6), which was associated with superior therapeutic efficacy. This enhanced fitness among vaccine-induced CD8^+^ TIL cells after skin immunization was also associated with spreading of the host T cell response to include recognition of melanoma Ags (gp100, TRP1) not included in the vaccine (figure 6), which we did not observe in mice treated with DC-based genetic vaccine. Hence, the efficacy of vaccines to best control multifocal disease in our melanoma models appears to be associated with the development of broadly reactive CD8^+^ T cell responses and enhanced IFNγ^+^CD8^+^ TIL survival. We hypothesize that skin immunization results in access to highly stimulatory dermal DC capable of priming highly fit type 1 CD8^+^ T cells that, on arrival in the TME, facilitate the secondary recruitment and activation of cross-presenting XCR1^+^DC^76–79^ and skin resident CD103^+^ DC. This then leads to corollary expansion in the CD8^+^ T cell repertoire in tdLN.

There is accumulating evidence supporting the translational/clinical importance of vaccine-induced, tumor Ag-specific CD4^+^ T cells in treatment outcomes.^80^ CD4^+^ T cells have also recently attracted attention in the context of effective αPD1-based intervention, despite a lack of consensus on their relevant mechanism(s) of action.^15–22^ In the current model, large numbers of CD4^+^ TIL appear to represent Treg-mediating enhanced suppressive activity.^62 69^ Skin vaccination appears to yield a less suppressive TME via reducing intratumoral Treg numbers, while coordinately increasing numbers of IL2^+^CD4^+^ T cells (figure 7H, I) predicted to be inherently more responsive to PD1 blockade (figures 2A and 4D). A new clinical study shows that a cancer neoantigen vaccine eliciting epitope spreading in combination with αPD1 treats advanced solid tumors.^81^ We are currently investigating this paradigm and its impact on treatment outcome in our checkpoint-resistant multifocal disease models using combination regimens including the skin immunization ± αPD1 checkpoint blockade.

Since Brafi treatment-resistant melanomas respond to skin immunization (figure 2B), we also plan to further investigate the therapeutic impact of Brafi treatment in combination with the skin immunization for possible translation of this approach into the clinic. In this light, it is important to note that combined treatment with Brafi, Meki and αPD1 has been recently shown to generate potent therapeutic responses in the transplantable SM1 (Braf^V600E^) melanoma model^11^ and that treatment of patients with advanced-stage Braf-mutant melanomas have displayed encouraging responses to treatment with this combination regimen.^82^ Hence, we also plan to investigate the therapeutic efficacy of skin immunization combined with Brafi+Meki in future studies.

In summary, we reported the xbp1/hsp70-driven TRP2 genetic skin immunization effectively promotes durable antitumor immunity against PD1 (and Brafi)-resistant, multifocal melanoma and that this treatment sensitizes PD1-resistant disease to corollary (re)treatment with αPD1 mAb-based immunotherapy. Although the defined mechanisms of action for effective skin immunization remain incompletely resolved, the therapeutic efficacy of this approach requires the participation of skin-resident cells in support of the development of broadly reactive antitumor CD8^+^ T cell responses, enhanced CD8^+^ TIL infiltration and fitness and improved levels of IL2^+^CD4^+^ TIL. We believe this novel skin immunization strategy has potential to more effectively treat melanoma patients with multifocal/disseminated disease in combination first-line interventional approaches or as a salvage approach after progression on αPD1-based and/or Brafi-based regimens.

## Materials and methods

### Mice

C57BL/6 (B6) wild type (WT) mice were purchased from Taconic (Rensselaer, New York) or JAX (Bar Harbor, Maine). B6-Tyr-Cre^ERT2^Braf^CA^Pten^lox/lox^ mice were described previously.^58 63 69^ All mice were housed, and B6-Tyr-Cre^ERT2^Braf^CA^Pten^lox/lox^ mice were bred in specific pathogen-free conditions in the University of Pittsburgh animal facility. Mice were used in experiments between the ages of 6–12 weeks, and B6-Tyr-Cre^ERT2^Braf^CA^Pten^lox/lox^ mice were used for induction of melanomas between the ages of 3 and 5 weeks, according to Institutional Animal Care and Use Committee (IACUC)-approved protocols and in accordance with recommendations for the proper use and care of laboratory animals.

### Vaccines

DNA vaccine (for skin immunization): plasmid DNA encoding xbp1 and hsp70 fused to TRP2 was described previously^57 58^ and was purified using EndoFree plasmid kits (Qiagen) following vendor’s instructions (Qiagen) (Valencia, California, USA). GG bullets were made from plasmid DNA (120 µg) and gold microcarriers (60 mg; 0.6 or 1 µm diameter; Bio-Rad) per the vendor’s instruction and stored at 4°C in the presence of desiccant pellets.

DC vaccine: DC were generated from BM of naïve B6 WT mice (male or female, 6–8 weeks) as reported previously.^57 58^ On day 6 of culture, CD11c^+^ DC (2×10^6^) purified from non-adherent cells using anti-CD11c microbeads (Miltenyi Biotec) (CD11c^+^ DC purity >95%) were transfected with 7 µg endotoxin-free vaccine DNA using an Amaxa mouse DC Nucleofector kit (Lonza) following the vendor’s instruction and cultured in RPMI 1640 media supplemented with 10%FBS, 2 mmol/L glutamine and 1× antibiotic/antimycotic solution without maturation factor(s). After 2 days, DC were gently collected and resuspended in endotoxic-free 1× PBS (Sigma) immediately prior to injection.

### Therapies

Braf mutant melanomas were induced by topical application of 4-hydroxytamoxifen (4HT) (H6278, Sigma) to male or female B6-Tyr-Cre^ERT2^Braf^CA^Pten^lox/lox^ mice.^58 63 69^ Melanomas also spontaneously develop in the skin of these animals due to ‘leaky’ Cre recombinase expression and activity on a genetic background favorable to tumor formation, which was carefully monitored.

Melanomas were allowed to progress to a mean tumor size of ~4 mm in diameter, at which time animals were randomized into cohorts exhibiting comparable mean tumor size. Mice were then left untreated or they were treated as follows with detailed time courses provided in individual figure legends (the day of first treatment was defined as day 0):

- DNA vaccine (four bullets per immunization per mouse) was cutaneously delivered using a GG with a Helios GG system (Bio-Rad) following the vendor’s instruction once (day 0) or three times weekly (days 0, 7 and 14) at the preshaved abdominal skin of mice.^58^ DC vaccine (2.5×10^5^ DC in 10–50 µL endotoxin-free 1× PBS) was delivered using an insulin syringe once (day 0) via intraperitoneal, i.d. or intratumoral;
- Brafi PLX4032 (ChemieTek, 50 mg/kg) or PLX4720 (Selleckchem, 30 mg/kg) was administered by oral gavage daily for 10 days;
- αPD1 (clone RPM114; BioXcell; 250 µg) was injected intraperitoneal every other day × 4; and
- Combinatorial treatment with DNA vaccine (A) and αPD1 (C).

Tumor incidence and growth were monitored every 2–3 days, with tumor size was estimated as the product of orthogonal measurements determined using a digital slide calipers (Fisher Scientific, Pittsburgh, Pennsylvania, USA). Mice were euthanized when the induced melanoma or the sum of multifocal disease (induced +spontaneous melanomas) reached 2 cm in mean diameter or when the mice exhibited distress due to metastatic disease (metastases are detected in dLN and lung; all induction treated mutant mice developed metastases in regional dLN).

### Prevention

B6-Tyr-Cre^ERT2^Braf^CA^Pten^lox/lox^ mice (male/female, 3–4 weeks) were vaccinated three times on a weekly basis (ie, days 0, 7 and 14, with the day of first treatment defined as day 0). On day 0, mice were topically treated with 4HT to induce local melanoma development. Tumor incidence, growth and animal overall survival was monitored as described above. Combinatorial treatment with DNA vaccine and αPD1 is described in figure legends.

### CD8^+^ T cell responses

Twenty-four days after the treatment, representative mice were intravenously injected with Brefeldin A (BFA; 250 µg/200 µL 1× Phosphate-buffered saline (PBS); Sigma, B-7651) for in vivo intracellular cytokine detection by flow cytometry.^83^ Four hours later, single cells from tdLN were preincubated in FACS staining buffer (1× PBS, 0.5% FBS, 2 mM EDTA) containing 1% Fc Block (BD Biosciences) on ice for 5 min and subsequently stained with anti-CD8-Alexa Flour700 (Biolegend) for 30 min on ice in the dark, and then washed, fixed and permeabilized with perm/fix buffer (eBioscience) and intracellularly stained with anti-IFNγ-PE (XMG1.2) (BD Biosciences) for 15 min on ice in the dark. At the same time of performing these experiments, single cell suspensions of TIL were obtained^69 84^ and stained with Fixable Viability Dye eFlour 780 (for excluding dead cells), anti-CD45-PerCP/Cy5.5, anti-CD8-Alexa Flour 700, anti-IFNγ-PE and anti-Caspases 3-BV605 (C92-605) (Invitrogen, BD Biosciences, Biolegend) as described above. After three final washes using FACS staining buffer, cells were resuspended in 500 µL 1% Paraformaldehyde (PFA) and analyzed by flow cytometry. Flow Data were acquired with an IMM Fortessa flow cytometer using BD FACSDiva software (BD Biosciences). Compensation was performed on the IMM Fortessa flow cytometer at the beginning of each experiment. Data were analyzed using FlowJo v10 (Treestar, Inc). In another set of experiments over the same time course, splenocytes (1×10^6^) from mice without injection of BFA were restimulated in 500 µL RPMI-1640 culture medium (CM) (10% FBS, 2 mmol/L glutamine and 1× antibiotic/antimycotic solution) in 48-well flat plates with 2 µg/mL of individual synthetic peptides: mouse TRP2_180–188_ (SVYDFFVWL), gp100_25-33_ (EGSRNQDWL), TRP1_222-229_ (TAYRYHLL), or OVA_257-264_ (SIINFEKL) as an Ag-specific control (University of Pittsburgh Peptide Synthesis Core; AnaSpec, Inc) at 37°C 5% CO_2_ for 3 days. The concentration of IFNγ in the culture supernatants was then measured using a cytokine-specific ELISA (BD Biosciences).

### Immunofluorescence (IF) staining

Fresh tumor tissues were harvested and immediately frozen in Scigen Tissue-Plus O.C.T. compound (Fisher Scientific) covered with liquid nitrogen cooled 2-Methylbutane (Sigma). Frozen samples were sectioned (7 µm) using a Cryostat at −20°C and mounted on Poly-L-lysine-coated adhesion glass slides (Thermo).

To perform IF staining, slides were fixed in PBS containing freshly prepared 2% PFA at room temperature (RT) for 20 min, and then washed three times for 5 min each with 1× PBS. Protein blocking was then performed using a blocking buffer (1× PBS with 1% BSA (Sigma), 0.3% Triton 100 (Sigma), 5% mouse serum (abcam), 5% Rat serum (STEMCELL), 5% goat serum (Jackson ImmunoResearch) and 2.4G2 (1/100) (BD Biosciences)) for 1 hour. This was followed by treatment using a AVIDIN/BIOTIN blocking kit (VECTOR). Sections were incubated with Avidin solution for 15 min. After rinsing with 1× PBS, sections were subsequently incubated with the biotin solution for 15 min. After washing with 1× PBS and then removing the blocking buffer by aspiration, antibodies diluted in perm buffer (1× PBS with 1% BSA, 0.3% Triton 100) were added and then incubated in humidified chamber overnight (16–18 hours) at 4°C. Samples were washed three times for 5 min with 1× PBS and an appropriate secondary species-specific antibodies were then incubated for 1 hour at RT in perm buffer. After washing in 1× PBS three times for 5 min, samples were mounted with mounting solution with DAPI (abcam) at RT for 5 min, before the application of a cover slip and storage at 4°C. The following antibodies were used in the staining: Alexa Fluor 647-CD45 (30-F11, 1/50, Biolegend), Alexa Fluor 488-CD45 (36-F11, 1/100, Biolegend), Alexa Fluor 488-CD4 (GK1.5, 1/100, Biolegend), Biotin-CD8 (53–6.7, 1/200, eBioscience), and Streptavidin-Cy3 (1:3000, Jackson ImmunoResearch).

Fluorescence images were captured using an ECLIPSE E800 microscope at 20× zoom and processed with SPOT software. DAPI (blue), CD4-AF488 (green) and CD45-AF647 (red) were displayed. In another panel, DAPI (blue), CD8-Cy3 (red) and CD45-AF488 (green) were displayed. Alternate adjustments were done in SPOT by modifying RGB histograms to sharpen the images. Finally, images were merged to show CD45^+^CD4^+^ T cells or CD45^+^CD8^+^ T cells as yellow. Quantification of T cells in tumor margin vs core was performed using ImageJ and Fiji software.

### Tumor-infiltrating T cells

Tumors were minced and digested with 1 mg/mL collagenase D, 1 mg/mL hyaluronidase and 1 mg/mL DNase (Sigma) in RPMI-1640 CM (10% FBS, 2 mM L-glutamine, 50 µM beta-mercaptoethanol, 1 mM sodium pyruvate, 1 mM non-essential amino acids, 1× antibiotic, 10 mM HEPES) and incubated at 37°C in a shaker at 250 rpm for 60 min. Cell suspensions were harvested by grinding and passage through a 70 µm cell strainer. After washing three times with 1× PBS, cells were cultured in a 48-well plate with 400 µL RPMI-1640 CM in the presence of 10 µg/mL Brefeldin A (BFA) for 4 hours. Surface and intracellular staining were performed using Fix & Perm Cell Permeabilization Kit (Life Technologies) and following antibodies: anti-CD45-PercpCy5.5 (30-F11, BD Biosciences), anti-CD4-BV750 (GK1.5, Biolegend), anti-CD8-BUV737 (53–6.7, BD Biosciences), anti-PD1-PE-Cy7 (J43, Invitrogen), and anti-IL2-BV421 (JES6-5H4, Biolegend). Dead cells were stained with Fixable Viability Dye (FVD) eFluor 780 at the same time. After three final washes using FACS staining buffer, cells were resuspended in 500 µL 1% PFA and analyzed by flow cytometry using an Aurora flow cytometer.

### Statistics

Immunological and tumor size data were statistically analyzed using Student’s t-test (GraphPad Prism, La Jolla, California, USA). Animal survival data were analyzed for statistical significance using log-rank test (GraphPad Prism) and presented in Kaplan-Meier survival curves. P<0.05 is considered to be statistically significant.

## References

[1] Ribas A, Wolchok JD Cancer immunotherapy using checkpoint blockade. Science 2018;359:1350–5. 10.1126/science.aar4060 29567705 PMC7391259

[2] Bollag G, Hirth P, Tsai J, et al Clinical efficacy of a RAF inhibitor needs broad target blockade in BRAF-mutant melanoma. Nature 2010;467:596–9. 10.1038/nature09454 20823850 PMC2948082

[3] Sharma P, Hu-Lieskovan S, Wargo JA, et al Primary, adaptive, and acquired resistance to cancer immunotherapy. Cell 2017;168:707–23. 10.1016/j.cell.2017.01.017 28187290 PMC5391692

[4] Villanueva J, Vultur A, Herlyn M, et al Resistance to BRAF inhibitors: unraveling mechanisms and future treatment options. Cancer Res 2011;71:7137–40. 10.1158/0008-5472.CAN-11-1243 22131348 PMC3588168

[5] Frederick DT, Piris A, Cogdill AP, et al BRAF inhibition is associated with enhanced melanoma antigen expression and a more favorable tumor microenvironment in patients with metastatic melanoma. Clin Cancer Res 2013;19:1225–31. 10.1158/1078-0432.CCR-12-1630 23307859 PMC3752683

[6] Wilmott JS, Long GV, Howle JR, et al Selective BRAF inhibitors induce marked T-cell infiltration into human metastatic melanoma. Clin Cancer Res 2012;18:1386–94. 10.1158/1078-0432.CCR-11-2479 22156613

[7] Steinberg SM, Zhang P, Malik BT, et al BRAF inhibition alleviates immune suppression in murine autochthonous melanoma. Cancer Immunol Res 2014;2:1044–50. 10.1158/2326-6066.CIR-14-0074 25183499 PMC4230697

[8] Ho P-C, Meeth KM, Tsui Y-C, et al Immune-Based antitumor effects of BRAF inhibitors rely on signaling by CD40L and IFNγ. Cancer Res 2014;74:3205–17. 10.1158/0008-5472.CAN-13-3461 24736544 PMC4063281

[9] Hu-Lieskovan S, Robert L, Homet Moreno B, et al Combining targeted therapy with immunotherapy in BRAF-mutant melanoma: promise and challenges. J Clin Oncol 2014;32:2248–54. 10.1200/JCO.2013.52.1377 24958825 PMC4164812

[10] Kwong LN, Boland GM, Frederick DT, et al Co-clinical assessment identifies patterns of BRAF inhibitor resistance in melanoma. J Clin Invest 2015;125:1459–70. 10.1172/JCI78954 25705882 PMC4396463

[11] Hu-Lieskovan S, Mok S, Homet Moreno B, et al Improved antitumor activity of immunotherapy with BRAF and MEK inhibitors in BRAF(V600E) melanoma. Sci Transl Med 2015;7:279ra4. 10.1126/scitranslmed.aaa4691 PMC4765379 25787767

[12] Wei SC, Duffy CR, Allison JP, et al Fundamental mechanisms of immune checkpoint blockade therapy. Cancer Discov 2018;8:1069–86. 10.1158/2159-8290.CD-18-0367 30115704

[13] Jenkins RW, Barbie DA, Flaherty KT, et al Mechanisms of resistance to immune checkpoint inhibitors. Br J Cancer 2018;118:9–16. 10.1038/bjc.2017.434 29319049 PMC5765236

[14] LaFleur MW, Muroyama Y, Drake CG, et al Inhibitors of the PD-1 pathway in tumor therapy. J Immunol 2018;200:375–83. 10.4049/jimmunol.1701044 29311378 PMC5924692

[15] Homet Moreno B, Zaretsky JM, Garcia-Diaz A, et al Response to programmed cell death-1 blockade in a murine melanoma syngeneic model requires costimulation, CD4, and CD8 T cells. Cancer Immunol Res 2016;4:845–57. 10.1158/2326-6066.CIR-16-0060 27589875 PMC5050168

[16] Klein S, Ziello J, Speranza M, et al PD-1 blockade activates conventional CD4 T cells and the innate immune response during glioblastoma eradication. J Immunol 2018;200 (1 Supplement) 57.9.

[17] Zappasodi R, Budhu S, Hellmann MD, et al Non-conventional inhibitory CD4^+^Foxp3^−^PD-1^hi^ T cells as a biomarker of immune checkpoint blockade activity. Cancer Cell 2018;33:1017–32. 10.1016/j.ccell.2018.05.009 29894689 PMC6648657

[18] Jiao S, Subudhi SK, Aparicio A, et al Differences in tumor microenvironment dictate T helper lineage polarization and response to immune checkpoint therapy. Cell 2019;179:1177–90. 10.1016/j.cell.2019.10.029 31730856

[19] Alspach E, Lussier DM, Miceli AP, et al MHC-II neoantigens shape tumour immunity and response to immunotherapy. Nature 2019;574:696–701. 10.1038/s41586-019-1671-8 31645760 PMC6858572

[20] Hao X, Falo III LD, Chen G, et al Blockading PD1 on tumor-primed CD4 T cells instigates antitumor immunity [abstract]. Cancer Immunol Res 2020;8 (3 Supplement) Abstract nr A37.

[21] Kagamu H, Kitano S, Yamaguchi O, et al CD4^+^ T-cell Immunity in the Peripheral Blood Correlates with Response to Anti-PD-1 Therapy. Cancer Immunol Res 2020;8:334–44. 10.1158/2326-6066.CIR-19-0574 31871122

[22] Oh DY, Kwek SS, Raju SS, et al Intratumoral CD4^+^ T Cells Mediate Anti-tumor Cytotoxicity in Human Bladder Cancer. Cell 2020;181:1612–25. 10.1016/j.cell.2020.05.017 32497499 PMC7321885

[23] Chow MT, Ozga AJ, Servis RL, et al Intratumoral activity of the CXCR3 chemokine system is required for the efficacy of anti-PD-1 therapy. Immunity 2019;50:1498–512. 10.1016/j.immuni.2019.04.010 31097342 PMC6527362

[24] Liu C, Peng W, Xu C, et al BRAF inhibition increases tumor infiltration by T cells and enhances the antitumor activity of adoptive immunotherapy in mice. Clin Cancer Res 2013;19:393–403. 10.1158/1078-0432.CCR-12-1626 23204132 PMC4120472

[25] De Henau O, Rausch M, Winkler D, et al Overcoming resistance to checkpoint blockade therapy by targeting PI3Kγ in myeloid cells. Nature 2016;539:443–7. 10.1038/nature20554 27828943 PMC5634331

[26] Salmon H, Idoyaga J, Rahman A, et al Expansion and Activation of CD103^(+^) Dendritic Cell Progenitors at the Tumor Site Enhances Tumor Responses to Therapeutic PD-L1 and BRAF Inhibition. Immunity 2016;44:924–38. 10.1016/j.immuni.2016.03.012 27096321 PMC4980762

[27] Sánchez-Paulete AR, Cueto FJ, Martínez-López M, et al Cancer immunotherapy with immunomodulatory Anti-CD137 and anti-PD-1 monoclonal antibodies requires BATF3-Dependent dendritic cells. Cancer Discov 2016;6:71–9. 10.1158/2159-8290.CD-15-0510 26493961 PMC5036540

[28] Ribas A, Dummer R, Puzanov I, et al Oncolytic virotherapy promotes intratumoral T cell infiltration and improves anti-PD-1 immunotherapy. Cell 2017;170:1109–19. 10.1016/j.cell.2017.08.027 28886381 PMC8034392

[29] Messenheimer DJ, Jensen SM, Afentoulis ME, et al Timing of PD-1 blockade is critical to effective combination immunotherapy with Anti-OX40. Clin Cancer Res 2017;23:6165–77. 10.1158/1078-0432.CCR-16-2677 28855348 PMC5641261

[30] Rech AJ, Dada H, Kotzin JJ, et al Radiotherapy and CD40 activation separately augment immunity to checkpoint blockade in cancer. Cancer Res 2018;78:4282–91. 10.1158/0008-5472.CAN-17-3821 29844122 PMC6415684

[31] Christmas BJ, Rafie CI, Hopkins AC, et al Entinostat converts Immune-Resistant breast and pancreatic cancers into Checkpoint-Responsive tumors by reprogramming tumor-infiltrating MDSCs. Cancer Immunol Res 2018;6:1561–77. 10.1158/2326-6066.CIR-18-0070 30341213 PMC6279584

[32] Sánchez-Paulete AR, Teijeira Álvaro, Quetglas JI, et al Intratumoral immunotherapy with XCL1 and sFlt3L encoded in recombinant Semliki Forest virus-derived vectors fosters dendritic cell-mediated T-cell Cross-Priming. Cancer Res 2018;78:6643–54. 10.1158/0008-5472.CAN-18-0933 30297531

[33] Ma HS, Poudel B, Torres ER, et al A CD40 agonist and PD-1 antagonist antibody reprogram the microenvironment of Nonimmunogenic tumors to allow T-cell-mediated anticancer activity. Cancer Immunol Res 2019;7:428–42. 10.1158/2326-6066.CIR-18-0061 30642833 PMC6397686

[34] Di Pilato M, Kim EY, Cadilha BL, et al Targeting the CBM complex causes T_reg_ cells to prime tumours for immune checkpoint therapy. Nature 2019;570:112–6. 10.1038/s41586-019-1215-2 31092922 PMC6656391

[35] Voorwerk L, Slagter M, Horlings HM, et al Immune induction strategies in metastatic triple-negative breast cancer to enhance the sensitivity to PD-1 blockade: the tonic trial. Nat Med 2019;25:920–8. 10.1038/s41591-019-0432-4 31086347

[36] Hammerich L, Marron TU, Upadhyay R, et al Systemic clinical tumor regressions and potentiation of PD1 blockade with in situ vaccination. Nat Med 2019;25:814–24. 10.1038/s41591-019-0410-x 30962585

[37] Knight DA, Ngiow SF, Li M, et al Host immunity contributes to the anti-melanoma activity of BRAF inhibitors. J Clin Invest 2013;123:1371–81. 10.1172/JCI66236 23454771 PMC3582139

[38] Carreno BM, Magrini V, Becker-Hapak M, et al Cancer immunotherapy. A dendritic cell vaccine increases the breadth and diversity of melanoma neoantigen-specific T cells. Science 2015;348:803–8. 10.1126/science.aaa3828 25837513 PMC4549796

[39] Zappasodi R, Merghoub T, Wolchok JD, et al Emerging concepts for immune checkpoint Blockade-Based combination therapies. Cancer Cell 2018;33:581–98. 10.1016/j.ccell.2018.03.005 29634946 PMC5896787

[40] Cancel J-C, Crozat K, Dalod M, et al Are conventional type 1 dendritic cells critical for protective antitumor immunity and how? Front Immunol 2019;10:9. 10.3389/fimmu.2019.00009 30809220 PMC6379659

[41] Ott PA, Wu CJ Cancer vaccines: steering T cells down the right path to eradicate tumors. Cancer Discov 2019;9:476–81. 10.1158/2159-8290.CD-18-1357 30862723 PMC7067230

[42] Yost KE, Satpathy AT, Wells DK, et al Clonal replacement of tumor-specific T cells following PD-1 blockade. Nat Med 2019;25:1251–9. 10.1038/s41591-019-0522-3 31359002 PMC6689255

[43] Li J, Byrne KT, Yan F, et al Tumor cell-intrinsic factors underlie heterogeneity of immune cell infiltration and response to immunotherapy. Immunity 2018;49:178–93. 10.1016/j.immuni.2018.06.006 29958801 PMC6707727

[44] Bettigole SE, Glimcher LH Endoplasmic reticulum stress in immunity. Annu Rev Immunol 2015;33:107–38. 10.1146/annurev-immunol-032414-112116 25493331

[45] Osorio F, Lambrecht BN, Janssens S, et al Antigen presentation unfolded: identifying convergence points between the UPR and antigen presentation pathways. Curr Opin Immunol 2018;52:100–7. 10.1016/j.coi.2018.04.020 29754111

[46] Cubillos-Ruiz JR, Silberman PC, Rutkowski MR, et al ER stress sensor XBP1 controls anti-tumor immunity by disrupting dendritic cell homeostasis. Cell 2015;161:1527–38. 10.1016/j.cell.2015.05.025 26073941 PMC4580135

[47] Song M, Sandoval TA, Chae C-S, et al IRE1α-XBP1 controls T cell function in ovarian cancer by regulating mitochondrial activity. Nature 2018;562:423–8. 10.1038/s41586-018-0597-x 30305738 PMC6237282

[48] Iwakoshi NN, Pypaert M, Glimcher LH, et al The transcription factor XBP-1 is essential for the development and survival of dendritic cells. J Exp Med 2007;204:2267–75. 10.1084/jem.20070525 17875675 PMC2118458

[49] Martinon F, Chen X, Lee A-H, et al TLR activation of the transcription factor XBP1 regulates innate immune responses in macrophages. Nat Immunol 2010;11:411–8. 10.1038/ni.1857 20351694 PMC3113706

[50] Nakaya HI, Wrammert J, Lee EK, et al Systems biology of vaccination for seasonal influenza in humans. Nat Immunol 2011;12:786–95. 10.1038/ni.2067 21743478 PMC3140559

[51] Hu F, Yu X, Wang H, et al ER stress and its regulator X-box-binding protein-1 enhance polyIC-induced innate immune response in dendritic cells. Eur J Immunol 2011;41:1086–97. 10.1002/eji.201040831 21400498 PMC3157298

[52] Savic S, Ouboussad L, Dickie LJ, et al TLR dependent XBP-1 activation induces an autocrine loop in rheumatoid arthritis synoviocytes. J Autoimmun 2014;50:59–66. 10.1016/j.jaut.2013.11.002 24387801 PMC4012140

[53] Osorio F, Tavernier SJ, Hoffmann E, et al The unfolded-protein-response sensor IRE-1α regulates the function of CD8α+ dendritic cells. Nat Immunol 2014;15:248–57. 10.1038/ni.2808 24441789

[54] Tavernier SJ, Osorio F, Vandersarren L, et al Regulated IRE1-dependent mRNA decay sets the threshold for dendritic cell survival. Nat Cell Biol 2017;19:698–710. 10.1038/ncb3518 28459443 PMC5563826

[55] Wang Y, Zhang Y, Yi P, et al The IL-15-AKT-XBP1s signaling pathway contributes to effector functions and survival in human NK cells. Nat Immunol 2019;20:10–17. 10.1038/s41590-018-0265-1 30538328 PMC6293989

[56] Dong H, Adams NM, Xu Y, et al The IRE1 endoplasmic reticulum stress sensor activates natural killer cell immunity in part by regulating c-myc. Nat Immunol 2019;20:865–78. 10.1038/s41590-019-0388-z 31086333 PMC6588410

[57] Tian S, Liu Z, Donahue C, et al Genetic targeting of the active transcription factor XBP1s to dendritic cells potentiates vaccine-induced prophylactic and therapeutic antitumor immunity. Mol Ther 2012;20:432–42. 10.1038/mt.2011.183 21934655 PMC3277233

[58] Zhang Y, Chen G, Liu Z, et al Genetic vaccines to potentiate the effective CD103^+^ dendritic cell-mediated cross-priming of antitumor immunity. J Immunol 2015;194:5937–47. 10.4049/jimmunol.1500089 25972487 PMC4458448

[59] Medel B, Costoya C, Fernandez D, et al IRE1a activation in bone marrow-derived dendritic cells modulates innate recognition of melanoma cells and favors CD8^+^ T cell priming. Front Immunol 2018;9:3050. 10.3389/fimmu.2018.03050 30687308 PMC6338037

[60] Malyshev I Immunity, tumors and aging: the role of Hsp70. Springer, 2013 10.1007/978-94-007-5943-5

[61] Spranger S, Bao R, Gajewski TF, et al Melanoma-intrinsic β-catenin signalling prevents anti-tumour immunity. Nature 2015;523:231–5. 10.1038/nature14404 25970248

[62] Steinberg SM, Shabaneh TB, Zhang P, et al Myeloid cells that impair immunotherapy are restored in melanomas with acquired resistance to BRAF inhibitors. Cancer Res 2017;77:1599–610. 10.1158/0008-5472.CAN-16-1755 28202513 PMC5380540

[63] Dankort D, Curley DP, Cartlidge RA, et al Braf(V600E) cooperates with Pten loss to induce metastatic melanoma. Nat Genet 2009;41:544–52. 10.1038/ng.356 19282848 PMC2705918

[64] Zhu J, Powis de Tenbossche CG, Cané S, et al Resistance to cancer immunotherapy mediated by apoptosis of tumor-infiltrating lymphocytes. Nat Commun 2017;8:1404. 10.1038/s41467-017-00784-1 29123081 PMC5680273

[65] Horton BL, Williams JB, Cabanov A, et al Intratumoral CD8^+^ T-cell Apoptosis Is a Major Component of T-cell Dysfunction and Impedes Antitumor Immunity. Cancer Immunol Res 2018;6:14–24. 10.1158/2326-6066.CIR-17-0249 29097422 PMC5754226

[66] Kansy BA, Concha-Benavente F, Srivastava RM, et al PD-1 Status in CD8^+^ T Cells Associates with Survival and Anti-PD-1 Therapeutic Outcomes in Head and Neck Cancer. Cancer Res 2017;77:6353–64. 10.1158/0008-5472.CAN-16-3167 28904066 PMC5690836

[67] Ma J, Zheng B, Goswami S, et al PD1^Hi^ CD8^+^ T cells correlate with exhausted signature and poor clinical outcome in hepatocellular carcinoma. J Immunother Cancer 2019;7:331. 10.1186/s40425-019-0814-7 31783783 PMC6884778

[68] Ishikawa M, Nakayama K, Nakamura K, et al High PD-1 expression level is associated with an unfavorable prognosis in patients with cervical adenocarcinoma. Arch Gynecol Obstet 2020;302:209–18. 10.1007/s00404-020-05589-0 32435885 PMC7266794

[69] Zhang Y, Tian S, Liu Z, et al Dendritic cell-derived interleukin-15 is crucial for therapeutic cancer vaccine potency. Oncoimmunology 2014;3:e959321. 10.4161/21624011.2014.959321 25941586 PMC4292719

[70] Sharma M, Fa’ak F, Janssen L, et al NKTR-214 enhances anti-tumor T cell immune responses induced by checkpoint blockade or vaccination. J Immunother Cancer 2017.

[71] Zingg D, Arenas-Ramirez N, Sahin D, et al The histone methyltransferase EZH2 controls mechanisms of adaptive resistance to tumor immunotherapy. Cell Rep 2017;20:854–67. 10.1016/j.celrep.2017.07.007 28746871

[72] Liu Z, Ge Y, Wang H, et al Modifying the cancer-immune set point using vaccinia virus expressing re-designed interleukin-2. Nat Commun 2018;9:4682. 10.1038/s41467-018-06954-z 30410056 PMC6224581

[73] Raeber ME, Rosalia RA, Schmid D, et al Interleukin-2 signals converge in a lymphoid–dendritic cell pathway that promotes anticancer immunity. Sci Transl Med 2020;12:eaba5464:561 10.1126/scitranslmed.aba5464 32938795

[74] Heath WR, Carbone FR The skin-resident and migratory immune system in steady state and memory: innate lymphocytes, dendritic cells and T cells. Nat Immunol 2013;14:978–85. 10.1038/ni.2680 24048119

[75] Pasparakis M, Haase I, Nestle FO Mechanisms regulating skin immunity and inflammation. Nat Rev Immunol 2014;14:289–301. 10.1038/nri3646 24722477

[76] Broz ML, Binnewies M, Boldajipour B, et al Dissecting the tumor myeloid compartment reveals rare activating antigen-presenting cells critical for T cell immunity. Cancer Cell 2014;26:638–52. 10.1016/j.ccell.2014.09.007 25446897 PMC4254577

[77] Spranger S, Dai D, Horton B, et al Tumor-residing Batf3 dendritic cells are required for effector T cell trafficking and adoptive T cell therapy. Cancer Cell 2017;31:711–23. 10.1016/j.ccell.2017.04.003 28486109 PMC5650691

[78] Brewitz A, Eickhoff S, Dähling S, et al CD8^+^ T Cells Orchestrate pDC-XCR1^+^ Dendritic Cell Spatial and Functional Cooperativity to Optimize Priming. Immunity 2017;46:205–19. 10.1016/j.immuni.2017.01.003 28190711 PMC5362251

[79] Böttcher JP, Bonavita E, Chakravarty P, et al Nk cells stimulate recruitment of cdc1 into the tumor microenvironment promoting cancer immune control. Cell 2018;172:1022–37. 10.1016/j.cell.2018.01.004 29429633 PMC5847168

[80] Borst J, Ahrends T, Bąbała N, et al CD4^+^ T cell help in cancer immunology and immunotherapy. Nat Rev Immunol 2018;18:635–47. 10.1038/s41577-018-0044-0 30057419

[81] Ott PA, Hu-Lieskovan S, Chmielowski B, et al A phase Ib trial of personalized neoantigen therapy plus anti-PD-1 in patients with advanced melanoma, non-small cell lung cancer, or bladder cancer. Cell 2020;183:347–62. 10.1016/j.cell.2020.08.053 33064988

[82] Dummer R, Lebbé C, Atkinson V, et al Combined PD-1, *BRAF* and MEK inhibition in advanced *BRAF*-mutant melanoma: safety run-in and biomarker cohorts of COMBI-i. Nat Med 2020;26:1557–63. 10.1038/s41591-020-1082-2 33020648

[83] Liu F, Whitton JL Cutting edge: re-evaluating the in vivo cytokine responses of CD8^+^ T cells during primary and secondary viral infections. J Immunol 2005;174:5936–40. 10.4049/jimmunol.174.10.5936 15879085

[84] Liu Z, Hao X, Zhang Y, et al Intratumoral delivery of tumor antigen-loaded DC and tumor-primed CD4^+^ T cells combined with agonist α-GITR mAb promotes durable CD8^+^ T-cell-dependent antitumor immunity. Oncoimmunology 2017;6:e1315487. 10.1080/2162402X.2017.1315487 28680744 PMC5486177

