# Supplementary material for: Skin immunization for effective treatment of multifocal melanoma refractory to PD1 blockade and Braf inhibitors
Source: J Immunother Cancer. 2021 Jan 6;9(1):e001179. doi: 10.1136/jitc-2020-001179 (PMC7789470; doi:10.1136/jitc-2020-001179)
Supplement: Supplementary data [file jitc-2020-001179supp001.pdf]

Supplemental Figure 1

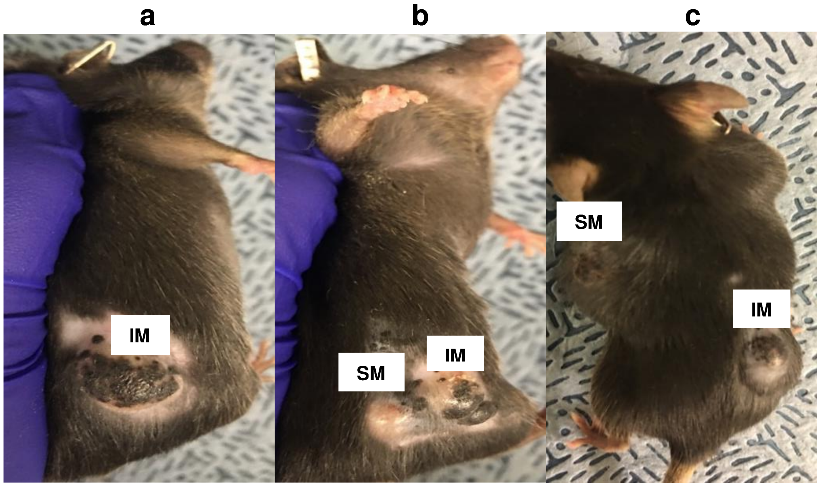

Supplemental Figure 1. Examples of induced vs. multifocal disease in Braf^{V600E}/Pten$^{-/-}$ mouse models. 4-hydroxytamoxifen was applied topically to B6-Tyr-Cre^{ERT2}Braf^{CA}Pten$^{lox/lox}$ mice (4-5 weeks), resulting (after 6-8 weeks) in the development of melanoma-bearing mice that could be clustered into 2 groups: an “induced” melanoma group presenting with tumors only at the site of tamoxifen application (a), and a multifocal disease group presenting with tumors at the site of tamoxifen administration as well as distal “spontaneous” tumors (b-c). IM: induced melanoma; SM: spontaneous melanoma.

Hao X, *et al. J Immunother Cancer* 2021; 9:e001179. doi: 10.1136/jitc-2020-001179

Supplemental Figure 2

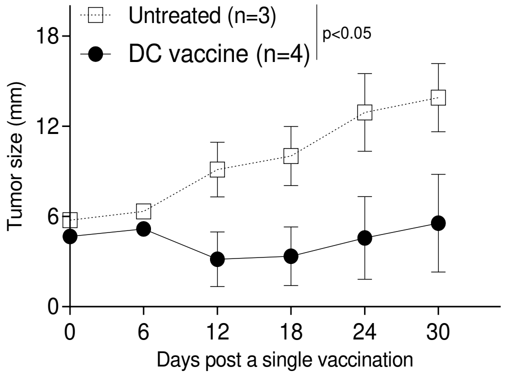

Supplemental Figure 2. BM-derived DC engineered with the vaccine construct cDNA and used to immunize mice controls the growth of s.c. GL26 glioma tumor. BM-derived DC engineered using the vaccine cDNA construct were used to vaccinate mice as described Figure 5. B6 mice bearing implanted s.c. GL26 glioma were left untreated or treated i.p. with the DC vaccine once. The growth of tumors was measured every 3 days. Data were statistically analyzed using a Student's t-test. n: numbers of mice used in experiments.

Hao X, *et al. J Immunother Cancer* 2021; 9:e001179. doi: 10.1136/jitc-2020-001179
